# Enteralgia—A Rare Case

**Published:** 1885-07-20

**Authors:** Johan S. Carothers

**Affiliations:** Mississippi


### ENTERALGIA.—A RARE CASE.

By JOHAN S. CAROTHERS, M. D., OF MISSISSIPPI.

On the 5th of March, 1885, I was called to see Mr. H. T. S., of this town, who has enjoyed reasonable health his entire life, save what was regarded occasional attacks of colic, at intervals of several years, supplemented with semi-occasional attacks of tic doloreux, the last being in the year 1876. On arrival, I found him suffering excruciating pain in the region of the rectum.

Inquiring into the history of the case, both as to its antecedents and present indications, found that constipation was habitual, and an existing torpidity indicated a cathartic treatment to unlock the bowels; and, accordingly, I administered a purgative, and, with it, tested anodynes to assuage the pain. Failing to give relief by any course pursued, I injected hypodermically 15 minims Majendie's solution; it, too, failing to bring relief, I bided my time for over an hour, my patient still agonizing in pain. I then administered, per orem, one grain sulphate morphia, and at once placed my patient in a sitting bath as hot as could be borne, removing the liquid as it cooled down, and replenishing with hot water—which afforded a partial relief, and that only temporary.

I this condition he passed the night and greater part of next day, being as long in the bath as out of it.

A short while after midnight, on the morning of the 6th, his bowels having acted freely, the pain became diffused over the whole bowels, while ever and anon a sharp twinge would shoot up into the chest, causing the most intense suffering, and interfering with respiration.

At this instance in the case the point of pain radiation, which had hitherto remained steadfastly in the rectum, now changed and took up position to the left of the umbilicus, near the great flexure of the colon, and there intensified the suffering, with occasional darts throughout the whole bowels.

So the day passed, with no intermission, or only of short duration, in the pain, and certainly no amelioration in the general condition of my patient, and, to my great surprise, no febrile reaction, the temperature and circulation remaining about normal.

In the night of the 6th, after a severe paroxysm of pain and dyspœna, there was a considerable eructation of gas from the stomach, with emission of mucous, when the situ of pain again shifted and located in a zone involving the diaphragm, and he commenced hiccoughing.

Hitherto I had apprehended invagination in the bowels, and my fears were greatly heightened by this new phase in the protean malady ; but the most careful examination failed to confirm my fears, and I assiduously, though tentatively, tried to palliate the distress.

The hiccoughs persisted despite the routine remedies—morphia hydrate chloral, chloroform, and the lesser anodynes, not excepting assafœtida and the much vaunted musk, and heroic counterirritation—four days and nights, and, together with the associate pain, found their own quietus through no interposition of ours, but solely at the behest of the conservative forces of nature, or probably was determinate and governed by time. Be this as it may, they both (the pain and the hiccough) pursued the even tenor of their way much to my dissatisfaction, and certainly to the great discomfort of my case, for four days and nights, in which he could scarcely eat or drink anything by which to sustain the overtaxed economy.

Now, the main point of interest in the case is not the peculiar and special situ of the neuralgia originally, but the protean phases it assumed in this: that it first appeared as a veritable neuralgia of the rectum, with a palpable engorgement of the same, as evinced by the fact that it was impossible to introduce a tube to any extent, and enemas introduced flowed back as fast as injected.

Again assuming the special phase of enteritis, by which we recognized it ; the special tenderness or rather augmentation of pain by pressure ; tymphanitis, tormina, and occasional mucous discharges, the clear cut symptoms by which that malady is known, save the non-existence of fever, inflammatory pulse, etc., which were wanting. Then the symptoms in the lower bowels subsiding and concentrating, as they did, in the left hypogastric region, and thence radiating over the entire bowels simulating ileus with its concomitant of pain, gaseous inflation, boborygmus, etc., wanting only in stercoraceous vomiting ; then invading the stomach, producing nausea, retching of mucus, etc., and, finally, attacking the diaphragm, producing an intractable hiccough, which, as before stated, lasted four days and nights. When it seemed that human endurance could bear no more, on the morning of the 10th, every symptom, as if by the magic of limitation solely, yielded the system to "tired nature's sweet restorer." After several hours' unbroken sleep, he awoke refreshed, his tormentor gone, with no visible trace of its ravages, if we except emaciation and an indescribable soreness over the entire bowels.

His restoration to health was rapid, although he could not bear any tonic treatment, i. e., quinine, iron ; stimulants were as fire to his bowels. In fact, at no time during his illness could he bear stimulants, though I much desired, and advised, their use, and they were repeatedly tried, only to aggravate his suffering.

His kidneys, on the first day of attack, were more or less affected, and acted sparingly, a dark, port wine-colored secretion, with a strong ammoniacal odor ; after which, they acted regularly and naturally, both as to appearance and quantity.

This analysis, although of necessity a little prolix, is as perfect a picture of the case as the space and circumstances would admit of, and is placed at your disposal with the hope, accompanied by request, that you publish.

Appealing to the known charity of the profession, especially of your readers, whose opinions I seek to know, I ask : Was I right in my diagnosis of the case, and what better line of practice could I have followed than the one vaguely indicated ?

The patient returned to his business duties in ten days.

